# Convergence of Ecohealth and One Health

**DOI:** 10.1007/s10393-013-0812-z

**Published:** 2013-02-08

**Authors:** Jakob Zinsstag

**Affiliations:** Department of Epidemiology and Public Health, Swiss Tropical and Public Health Institute, PO Box 4002, Basel, Switzerland

Interest and participation in EcoHealth and One Health approaches have grown considerably over the past decade. Clearly, the two approaches have many similarities and strive for similar outcomes, and collaboration between them holds great potential value. Convergence of the two approaches could avoid repetition, strengthening both communities yet still highlighting the unique attributes of each approach. In 2011 Margot Parkes, then President of the International Association for Ecology and Health (EcoHealth), called for critical reflection on directions for ecohealth as a transdisciplinary field positioned among converging efforts (Parkes 2012). Building on this, seventeen of us engaged in further discussion about the convergence between One Health and Ecohealth at the 4th biennial EcoHealth conference this past October. We represent veterinary medicine, public health, ecology and several other disciplines, geographic locations, and both Ecohealth and One Health experiences. We present our deliberations and insights on two questions: Where and how can the two concepts converge? What is their common ground and where can they live happily apart?

## Of Ecohealth and One Health

Several conceptual and foundational papers on One Health and Ecohealth frame our discussion. Ecohealth, as represented by the IAEH, strives for sustainable health of people, animals, and ecosystems by promoting discovery and understanding through transdisciplinary action-research. It encourages problem solving that draws upon multiple types of knowledge from the natural, social and health sciences, and the humanities (IAEH ratified constitution 2008, www.ecohealth.net). Others have documented the evolution of the field of ecohealth (Parkes 2012 in this journal). Still others have focused on elucidating principles fundamental to engaging in ecohealth research in a development context, for example: a systemic approach, tackling complex, non-linear problems quantitatively and qualitatively; a transdisciplinary approach that engages stakeholder participation; attention to environmental sustainability, and to gender and social equity; and transforming knowledge into action (Charron 2012).

The contemporary One Health approach recognises that the health of humans, animals, and ecosystems is intimately connected and involves a coordinated, collaborative, interdisciplinary, and cross-sectoral approach to addressing a wide range of risks at the animal–human–ecosystem interface. Embracing the Manhattan principles (Cook et al. 2004), the One Health movement strives to develop new methods to assess human and animal health and their respective social and environmental determinants and to assess the added value of improved cooperation and collaboration of human and animal health disciplines, research and interventions (Enserink 2007; Zinsstag et al. 2011, 2012).

## Exploring Common Ground and Unique Attributes

The Ecohealth and the One Health movement both emphasize an holistic understanding of health beyond the purely biomedical. They both champion systems thinking as a way of achieving a greater understanding of health problems, and both espouse inter- and trans-disciplinary research and collaborative participation. Areas already well developed in Ecohealth, such as the relationships between health and ecosystems or between health and sustainable development, have been growing in importance within the One Health movement, particularly in zoonosis control and pastoralist health. Both fields do not yet have strong plant health involvement, but this is just beginning. Ecohealth emerged from research oriented towards understanding health in the context of ecosystems, environmental degradation and unsustainable development. It presupposes that human survival depends on healthy and diverse ecosystems, and that both are under threat. Advocates and practitioners of One Health can build on the experiences of Ecohealth to strengthen these aspects in One Health research and interventions.

One Health is a movement that has its origins in the management of the disease threats to humans and animals. Its focus was primarily on the interlinkages between human and animal health and more recently on how these link to and impinge on the environment. Animal health is well within the scope of Ecohealth (for example, wildlife health and ecosystem change; various zoonoses of poverty), but not its focus. Ecohealth could further enhance these efforts by drawing on the One Health expertise and experiences in zoonotic disease research, and disease economics, surveillance and management.

While One Health is growing in disciplinary diversity, veterinarians and public health practitioners currently form the majority of the field, and it tends to focus on communicable disease, food safety, nutrition and antimicrobial resistance, issues squarely at the nexus of human and animal health. There are gains to be made in synergizing with the already-diverse membership in Ecohealth. The threat of a global influenza pandemic gave great impetus to the One Health movement, and was able to influence the World Organization for Animal Health (OIE), the Food and Agriculture Organization (FAO) and the World Health Organization (WHO) to formally work together under the banner of One Health. Similar collaborations were established in many countries, influencing national policies, investments and research funding. Ecohealth affiliates could learn from the strategies and networks used by One Health advocates to widen their influence on large-scale policy processes.

One Health has chosen not to have an organisational structure, a single journal for research or policy dissemination, or undertake regular conferencing arrangements. By partnering with existing structures such as the IAEH and its journal, *EcoHealth,* and with communities of practice in Ecohealth around the world, individuals who identify with the One Health movement gain new platforms for interaction and outreach.

## Looking Forward

Ecohealth and One Health share many values and approaches, and converge most obviously in the areas of zoonoses, disease emergence, and pandemic threats. Each has strengths to offer the other and by working together, greater impact may be achieved in global health and sustainability. Each also embraces themes and approaches not currently suited to the other. These differences should not prevent a closer interaction; rather, both communities stand to gain from shared information, resources and collaborative action. For example, Ecohealth, with its foundations in environmental issues, could contribute to the enhanced development of ecosystem approaches in One Health. Similarly, One Health could bolster the animal health research in EcoHealth as well as strengthen its economic evaluations and policy influence.

Some organizations are already employing the strengths of both approaches. For example, the Centre Suisse de Recherches Scientifiques in Côte d’Ivoire uses One Health approaches for diseases at the human-animal interface or food borne disease, and Ecohealth approaches for research on climate change, water and vector borne diseases.

Multiple benefits may be gained by bringing together One Health and Ecohealth, yet many people working in one or the other field are unaware of the potential for collaboration. Opportunities abound for reversing this: for example, through joint conferences, shared training, and publication in journals read by both communities of researchers, such as *EcoHealth*.

## Participants in the Symposium and Contributors

Jakob Zinsstag (convener), Swiss Tropical and Public Health Institute, University of Basel, Switzerland

Martyn Jeggo (convener), Australian Animal Health Laboratory, CSIRO, Australia

Esther Schelling, Swiss Tropical and Public Health Institute, Basel, Switzerland

Bassirou Bonfoh, Centre Suisse de Recherches Scientifiques en Côte d’Ivoire

David Waltner-Toews, University of Guelph, Ontario, Canada

Stefano Lelii, Institute of Public Health, Ruprecht-Karls-University, Heidelberg, Germany

Bob Williams, Wellington, New Zealand

Mahamat Bechir, Centre de Support en Santé Internationale, Chad

Felix Li, Public Health Agency of Canada, Canadian Embassy, China

Dominique Charron, International Development Research Center, Ottawa, Canada

Robinson Mdegela, Sokoine University of Agriculture, Tanzania

Meredith Barrett, Robert Wood Johnson Foundation Health and Society Scholars, University of California, San Francisco and University of California, Berkeley, CA, USA

Richard Kock, Royal Veterinary College, Hertfordshire, United Kingdom

Ibrahima Sy, Swiss Tropical and Public Health Institute, Basel, Switzerland

Alain Vandersmissen, European Union - European External Action Service, Brussels, Belgium

Peter Black, Department of Agriculture, Fisheries and Forestry, Barton, ACT, Australia

Peter Daszak, EcoHealth Alliance, New York, USA

## References

[CR1] Charron DF (2012). Ecosystem approaches to health for a global sustainability agenda. EcoHealth.

[CR2] Cook RA, Karesh WB, Osofsky SA (2004). The Manhattan Principles on ‘One World One Health’.

[CR3] Enserink M (2007). Medicine. Initiative aims to merge animal and human health science to benefit both. Science.

[CR4] Parkes M (2012) Diversity, emergence, resilience: Guides for a new generation of ecohealth research and practice. EcoHealth 8(2). doi:10.1007/s10393-011-0732-8. 10.1007/s10393-011-0732-8 22231862

[CR5] Zinsstag J, Mackenzie JS, Jeggo M, Heymann DL, Patz JA, Daszak P (2012). Mainstreaming one health. EcoHealth.

[CR6] Zinsstag J, Schelling E, Waltner-Toews D, Tanner M (2011). From “one medicine” to “one health” and systemic approaches to health and well-being. Preventive Veterinary Medicine.

